# B Cell Subsets and Cellular Signatures and Disease Relapse in Lupus Nephritis

**DOI:** 10.3389/fimmu.2020.01732

**Published:** 2020-09-10

**Authors:** Desmond Y. H. Yap, Susan Yung, Paul Lee, Irene Y. L. Yam, Cheryl Tam, Colin Tang, Tak Mao Chan

**Affiliations:** Division of Nephrology, Department of Medicine, Queen Mary Hospital, The University of Hong Kong, Pok Fu Lam, Hong Kong

**Keywords:** B cells, subsets, B cell signatures, lupus nephritis, disease relapse

## Abstract

**Introduction:**

Renal relapses adversely affect the long-term outcomes of patients with lupus nephritis (LN), but the pathogenic mechanisms remain elusive. B cell signatures of miR-148a, BACH1, BACH2, and PAX5 expression are relevant to the regulation of B lymphocyte homeostasis. It is unknown whether B cell signature is related to the relapse of LN.

**Methods:**

We compared B lymphocyte subsets and cellular signatures during disease quiescence between LN patients with multiple relapses (MR, ≥3 LN relapses within 36 months) and those with no relapse (NR). Also, circulating B lymphocytes were isolated from treatment-naïve patients with active LN and treated with antagomir-148a *in vitro* to investigate the relationship between miR-148a, BACH1, BACH2, and PAX5.

**Results:**

MR patients (*n* = 19), when compared with NR (*n* = 14), showed significantly lower percentage of circulating naïve B cells and higher memory B cell-to-naïve B cell ratio. MR patients also showed higher miR-148a levels in sera and B cells, and lower BACH1, BACH2, and PAX5 expression in naïve and memory B cells. Antagomir-148a upregulated BACH1, BACH2, and PAX5 expression, and reduced B cell proliferation upon stimulation, in naïve and memory B cells isolated from treatment-naïve active LN patients.

**Conclusion:**

Altered B cell subsets and cellular signatures of miR-148a, BACH1, BACH2, and PAX5 may be associated with distinct patient phenotypes related to the risk of LN relapse.

## Introduction

Lupus nephritis (LN) is a common and severe organ involvement in patients with systemic lupus erythematosus (SLE). Although the use of effective immunosuppressive treatments have markedly improved the clinical outcomes of LN patients (1, 2), disease relapse still constitutes a clinically important issue in the management of LN patients. Repeated LN relapses will lead to attrition of nephrons and increased cumulative toxicities of immunosuppressive medications, thus jeopardizing the long-term patient and renal outcomes (3–7).

The mechanisms leading to disease relapse in LN remain elusive, which renders the prediction and prevention of relapse clinically challenging. Aberrant lymphocyte response, breach of B cell tolerance, and hyper-reactivity of B lymphocytes all contribute to the pathogenesis of LN (8, 9). Both B and T cells exhibit immunological memory, a property which allows lymphocytes to react efficiently to autoantigens that they have been exposed to previously. Previous studies also demonstrated that memory B cells and plasma cells are less affected by conventional immunosuppressive treatments and hence are more readily reactivated resulting in disease relapse (10, 11). While both B and T lymphocytes serve as crucial effector immune-reactive cells, the B cell repertoire is believed to play an important role in LN relapse, as renal relapse is often correlated with or preceded by, a rise in autoantibodies such as anti-dsDNA. Indeed, perturbations in B lymphocyte subpopulations have been noted in lupus patients during different disease activity states (10, 12).

The B cell repertoire is regulated by various microRNAs and B cell transcription factors. In this context, microRNA-148a (miR-148a) is highly abundant in B cells and plasma cells, and has been shown to control important B cell transcription factors (e.g., BACH2) in promoting plasma cell differentiation and regulating B cell tolerance (13, 14). Upregulation of miR-148a can decrease *Gadd45a*, *Pten*, and *Bcl2l11* expressions, and thereby inhibit the apoptosis of immature B lymphocytes upon B cell receptor engagement (14). Moreover, the maturation and proliferation of B lymphocytes and plasma cells are orchestrated by important B cell transcription factors such as BACH1, BACH2, and PAX5 (15, 16). BACH2, with BACH1 serving as an auxiliary, shows critical functions in various stages of B cell development. BACH2 together with BACH1 suppresses the “myeloid genes” in pre-pro-B cells by binding to their putative regulatory regions, and promotes early B cell development (15). BACH2 also helps to determine B cell subpopulations within germinal centers, and can interact with BCL-6 to inhibit Blimp-1 transcription and thus plasma cell differentiation (17). Previous studies also reported that murine splenic B lymphocytes, in the absence of BACH2, showed increased differentiation into plasma cells via both Blimp-1-dependent and -independent pathways (18). PAX5 is a pivotal regulator in B cell development as the differentiation and functions of all mature B lymphocytes are highly dependent on PAX5 expression. PAX5 directs lymphoid progenitor cells to commit to the B cell lineage, promotes B lymphocytes maturation, and also regulates V(H)-DJ(H) recombination during antibody synthesis (16). Taken together, downregulation of transcription repressors BACH2, BACH1, and PAX5 are instrumental for normal homeostasis of B lymphocytes and plasma cells, and aberrant expression of these transcription factors have been implicated in the development of autoimmune and hematological disorders (15, 16). Furthermore, the homeostasis and function of lymphocytes are also influenced by the cytokine milieu. In this context, BAFF, IL-6, and IL-21 affect B cell survival and differentiation while IL-2, IL-4, IL-6, IL-10, IL-18, IFN-α, IFN-γ, IL-17, IL-21, and IL-23 can modulate Th1/Th2 and Th17/Treg balance, and elevated levels of these cytokines have been observed in SLE, including patients with LN (19–27). While these B cell signatures and cytokines have important regulatory effects on B lymphocyte biology, their roles and changes in LN relapse have not been fully elucidated.

Based on these backgrounds, we hypothesize that altered B cell subsets and related cellular signatures may be associated with differences in the risk of disease relapse in LN. In this study we examined B lymphocyte subsets, levels of related cytokines, and B cell signatures in LN patients during disease quiescence, and compared two distinct clinical phenotypes characterized by multiple relapses (MR) or no relapse (NR) after initial presentation. We also performed *in vitro* studies with B cells isolated from treatment-naïve active LN patients to investigate the effect of miR-148a inhibition on BACH1, BACH2, and PAX5 expression and cell proliferation.

## Materials and Methods

### Patients

The study was approved by the Institutional Review Board of the University of Hong Kong/Hospital Authority Hong Kong West Cluster (Approval number: UW 12-389). All experiments in this study followed the general requirements specified by the work safety regulations approved by the University of Hong Kong, which was in accordance with good practices and standards along the lines of CEN15793:2011 and WHO guidelines in biosafety and biosecurity. To compare the lymphocyte subsets, serum cytokines, and B cell signatures in MR and NR patients, blood samples (30 ml) were obtained from biopsy-proven Class III/IV ± V LN patients with the following inclusion criteria: (1) patients who had multiple relapses (defined as ≥3 LN relapses within 36 months, unrelated to treatment non-compliance) (MR group) or those with no relapse (defined as never relapsed after the first episode of nephritis) (NR group); and (2) patients with quiescent disease (SLEDAI score <4 with no points in the renal domain), and on a stable dose of prednisolone (5–7.5 mg/day for ≥4 months) alone or in combination with mycophenolate (1–1.5 g/day) or azathioprine (50–100 mg/day) as maintenance treatment. LN relapse was defined as proteinuria >1 g/day, presence of urinary red blood cells (RBC) >30/hpf or RBC casts, a 15% increase in serum creatinine compared with baseline, and anti-dsDNA level >30 IU/ml. LN relapse was confirmed with a kidney biopsy. Exclusion criteria were: (1) patients who received calcineurin inhibitors or mammalian target of rapamycin inhibitors as maintenance immunosuppression, or biologics (e.g., rituximab, belimumab, or abatacept) in the preceding 12 months; (2) patients who relapsed due to treatment non-compliance.

For the antagomir studies, we obtained blood samples (30 ml) from treatment-naïve patients with biopsy-proven Class III/IV ± V LN and active renal disease (denoted by proteinuria >1 g/day, presence of urinary RBC >30/hpf or RBC casts, a 15% increase in serum creatinine compared with baseline, and anti-dsDNA level >30 IU/ml). Blood samples (30 ml) were also obtained from healthy subjects as control.

### Analysis of Lymphocyte Subsets

Peripheral blood mononuclear cells (PBMC) were isolated using Lymphoprep^TM^, then washed with PBS and resuspended in 1 ml RPMI 1640 medium supplemented with 10% FCS, 10% DMSO, and 1% penicillin/streptomycin. PBMC were washed with PBS and stained with Zombie NIR fixable viability dye (diluted 1:100) for 30 min at room temperature. Non-specific Fc binding was blocked with Human TruStain FcX solution for 15 min on ice. For analysis of B lymphocyte subsets, cells were stained with monoclonal antibodies against human CD20 (1:40), together with CD27 (1:40), and CD138 (1:5) followed by incubation on ice for 30 min. For the assessment of T lymphocyte subsets, aliquots of PBMC were activated with phorbol myristate acetate (PMA, 50 ng/ml) and calcium ionophore (0.5 μg/ml) for 4 h in a 37°C tissue culture incubator. Brefeldin A (5 μg/ml) was added to samples during the last 3 h of incubation with PMA and calcium ionophore. PBMC were washed with staining buffer (PBS with 5% EDTA and 0.5% BSA) and stained with Zombie NIR fixable viability dye (diluted 1:100) for 30 min at room temperature. Non-specific Fc binding was blocked as above. Cells were incubated with monoclonal antibodies against CD25 (1:40) for 30 min on ice, then fixed and permeabilized. Cells were then labeled with monoclonal antibodies against CD3 (1:40) and/or CD4 (1:20), CD8 (1:40), IFN-γ (1:200), IL-4 (1:250), IL-17 (1:10), and FoxP3 (1:40) for 50 min on ice. Stained lymphocytes were washed twice with staining buffer prior to analysis using a BD LSRFortessa^TM^ flow cytometer (BD Biosciences) and 50,000 cells counted. Data were analyzed using FlowJo software Version 10 (Tree Star Inc., Ashland, OR, United States). The percentage of naïve B cells (CD20^+^CD27^–^), memory B cells (CD20^+^CD27^+^), plasma cells (CD20^–^CD27^+^CD138^+^), cytotoxic T cells (CD3^+^CD8^+^), Th1 cells (CD3^+^CD4^+^IFN-γ^+^), Th2 cells (CD3^+^CD4^+^IL-4^+^), Th17 cells (CD3^+^CD4^+^IL-17^+^), and Treg (CD3^+^CD4^+^CD25^+^FOXP3^+^) were analyzed and compared between MR and NR patients.

### Measurements of Serum Cytokine Levels

Serum levels of cytokines were determined by commercially available ELISA kits according to manufacturers’ instructions (BAFF, IL-10, and IL-18 by Quantikine^TM^ ELISA kits, R&D Systems Inc., Bio-Techne H.K. Limited, Hong Kong; IL-2, IL-4, IL-6, IL-17, and IFN-γ by MiniABTS ELISA Development kits, PeproTech, Dakewe BioTech (H.K.), Hong Kong; IL-21 and IL-23 by Ready-Set-Go! ^TM^ ELISA kit, ThermoFisher Scientific, Life Technologies Limited, Hong Kong; and IFN-α by ELISA^*PRO*^ kit, Mabtech, Dakew BioTech (H.K.), Hong Kong). The detection limits for BAFF, IL-2, IL-4, IL-6, IL-10, IL-17, IL-18, IL-21, IL-23, IFN-α, and IFN-γ were 62.5–4,000 pg/ml, 62.5–4,000 pg/ml, 15.6–1,000 pg/ml, 23.0–1,500 pg/ml, 0.78–50 pg/ml, 15.6–1,000 pg/ml, 25.6–1,000 pg/ml, 8.0–1,000 pg/ml, 15.0–2,000 pg/ml, 3.16–316 pg/ml, and 23.0–1,500 pg/ml respectively.

### Isolation of Memory and Naïve B Cells for *in vitro* Studies

PBMC were first separated from whole blood (30 ml) obtained from LN patients or healthy subjects using Lymphoprep^TM^, and naïve and memory B cells were further isolated by the EasySep Human Memory B Cell Isolation Kit (STEMCELL^TM^ Technologies, Lokco Technology Limited, Hong Kong) according to manufacturer’s instructions using 1 × 10^8^ cells/ml in a 1 ml suspension. The purity of isolated B cells was 96.7–98.0% as confirmed by flow cytometry.

### *In vitro* Antagomir-148a Studies

**FIGURE 1:**
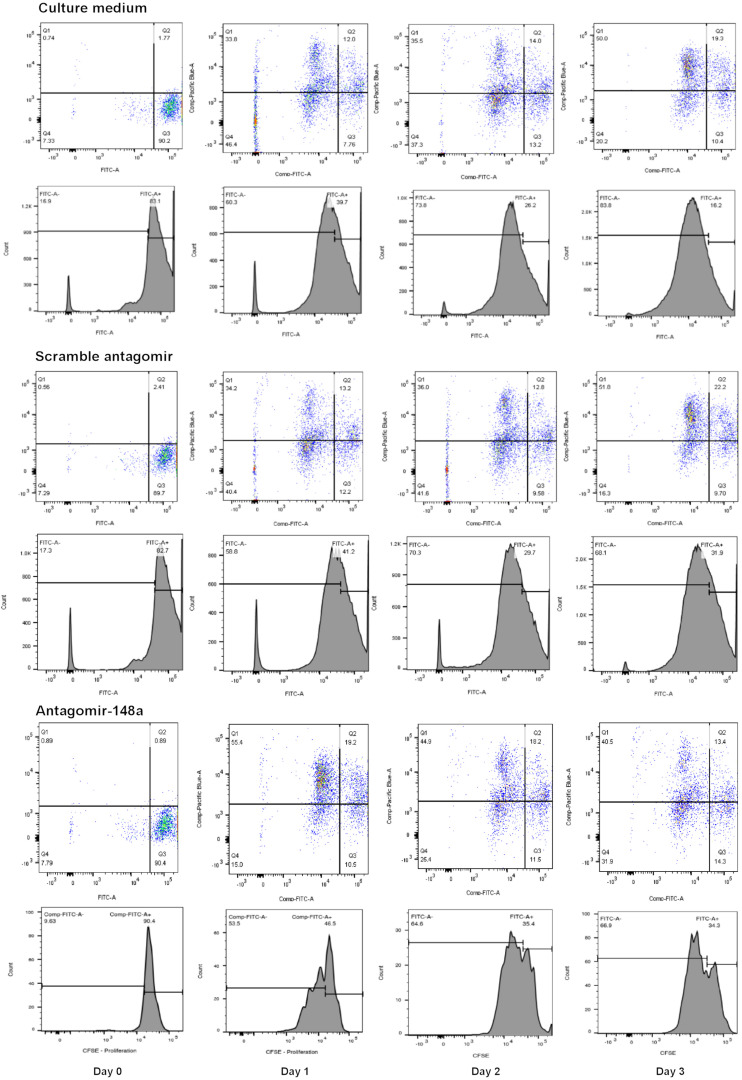
A representative flow cytometric analysis of the CSFE proliferation assay for B cells treated with scramble antagomir or antagomir-148a.

To determine the effect of miR-148a on cell proliferation and BACH1, BACH2, and PAX5 gene expression, isolated B cells from treatment-naïve LN patients with active disease were plated at a density of 10^5^ cell/ml in 96-well plates (100 μl/well) to assess changes in cell proliferation or in 24-well plate (1 ml/well) for total RNA extraction. For cell proliferation studies, B lymphocytes were labeled with carboxyfluorescein succinimidyl ester (CSFE, 5 μM in PBS final concentration) for 30 min prior to stimulation. B lymphocytes were pre-incubated with or without, scrambled antagomir (SCr-antagomir, as control) (1 μM) or antagomir-148a (1 μM) for 2 h, and then stimulated with CpG (2.5 μg/ml) in RPMI 1640 medium containing 10% FCS and 1% penicillin/streptomycin for up to 3 days (28). Synthetic antagomirs (2-*O*-methyl RNA oligo) targeting miR-148a (sequence: 5-mA(^∗^)mC(^∗^)mAmAmAmGmUmUmCmUmGm UmAmGmUmGm CmAmC(^∗^)mU(^∗^)mG(^∗^)mA(^∗^)-3-Chol) and scramble control (sequence: 5-mU(^∗^)mC(^∗^)mAmCmGmCmA mGmAmUmUmcMAmUmAmA(^∗^)mC(^∗^)mG(^∗^)mU(^∗^)-3-Chol) were custom-synthesized by Applied Biological Materials Inc. (Lokco Technology Limited, Hong Kong). All ribonucleotides are 2′-*O*-methyl modified (mN) and (^∗^) represent a phosphorothioate modification of the backbone. At the 3′-end of the oligonucleotides, a cholesterol molecule was added. Cell proliferation was assessed every 24 h for up to 72 h, and total RNA was extracted after 3 days of stimulation. The cell proliferation of naïve and memory B cells was determined by CSFE signals using BD LSRFortessa^TM^ flow cytometer (BD Bioscience) (Figure 1), and expressed as a percentage of total cells counted (50,000 cells).

### Determination of miR-148a, BACH1, BACH2, and PAX5 in Sera and B Cells

Total RNA was extracted from B lymphocytes isolated from LN patients and healthy controls, and also from cultured B cells following incubation with antagomir-148a as stated above. microRNAs were extracted from sera using the miRVANA^TM^ PARIS^TM^ kit (Applied Biosystems, Life Technologies Limited, Hong Kong) according to the manufacturer’s instructions. The levels of miR-148a, BACH1, BACH2, and PAX5 were determined by quantitative qPCR using standard methods. miR-148a expression was normalized to U6 expression (both measured by Taqman miRNA assay; Assay 000470 for miR-148a and Assay 001973 for U6). BACH1, BACH2, and PAX5 expressions were normalized to GAPDH expression. The primer sequences of BACH1, BACH2, PAX5, and GAPDH were listed as follows:

**Table:** 

**Gene**	**Direction**	**Sequence (5′ to 3′)**
BACH1	Forward	5′-TAG TGT GGA GCG AGA AGT GG-3′
	Reverse	5′-ACC TAA CCA CGG ACA CTC AG-3′
BACH2	Forward	5′-CCA GCA ATG ACT CAG GCA TC
	Reverse	5′-TCA TGA GTC TTG TCG CTG GT-3′
PAX5	Forward	5′-GGG TGG AGT GGG AGA AAT CA-3′
	Reverse	5′-CCA TGT TCT CTG GTT CCC CT-3′
GAPDH	Forward	5′-TGA CCT TTC TGT AGC TGG GG-3′
	Reverse	5′-CAA GCC CAC CCC TTG TCT AA-3′

### Statistical Analysis

Categorical variables were expressed as frequencies (percentages), and analyzed by the Chi-square or Fisher-Exact test where appropriate. Continuous variables were expressed as mean ± SD or median (range), and compared by the Mann-Whitney test. Inter-group comparisons were analyzed by ANOVA, followed by a *post hoc* Dunnett’s test. Associations between different clinical and immunological parameters were assessed by Spearman rank’s correlation coefficient. All statistical analyses were performed using a Graphpad Prism 7.0 (San Diego, CA, United States) and p values of less than 0.05 were considered statistically significant.

## Results

### Patient Characteristics

Circulating lymphocyte subsets, serum cytokine levels, and B cell signatures were analyzed in 33 LN patients (19 patients in the MR group and 14 patients in the NR group, respectively) during disease quiescence. Patient characteristics are presented in Table 1.

**TABLE 1:** Clinical characteristics of lupus nephritis patients with multiple relapses or those with no relapse and those with active nephritis.

	**MR (*n* = 19)**	**NR (*n* = 14)**	**Active LN (*n* = 8)**	***p* value^†^**
Sex (F/M)	16/3	14/0	5/3	0.244
Age (years)	48.0 ± 6.3	52.1 ± 9.6	38.0 ± 19.5	0.240
Class of LN				
III	4	6	0	0.260
IV	5	7	6	0.270
III + V	4	0	1	0.120
IV + V	6	1	1	0.090
Duration of follow-up from last nephritic episodes (months)	81.8 ± 46.7	110.9 ± 21.3	25.4 ± 17.7	0.091
Maintenance treatment				
PRED alone (*n*, %)	4 (21.0%)	6 (42.8%)	2 (25.0%)	0.257
PRED + MMF (*n*, %)	11 (57.9%)	4 (28.6%)	6 (75.0%)	0.158
PRED + AZA (*n*, %)	4 (21.1%)	4 (28.6%)	0 (0%)	0.442
Clinical parameters				
White cell count (×10^9^/ml)	5.4 ± 1.9	4.9 ± 1.6	5.2 ± 2.4	0.480
Lymphocyte count (×10^9^/ml)	1.3 ± 0.5	1.4 ± 0.7	0.9 ± 0.4	0.951
Serum C3 (mg/dl)	94.9 ± 31.7	80.0 ± 19.9	43.5 ± 22.8	0.329
Anti-dsDNA (IU/ml)	34.8 ± 38.8	23.9 ± 21.5	245.5 ± 101.4	0.910
eGFR (ml/min/1.73 m2)	59.4 ± 27.9	74.3 ± 21.2	61.3 ± 31.2	0.107
Serum albumin (g/l)	39.8 ± 5.5	43.1 ± 2.1	27.4 ± 5.8	0.055
Urine protein excretion (g/D)	0.28 ± 0.38	0.06 ± 0.11	3.2 ± 2.8	0.238

*AZA, azathioprine; eGFR, estimated glomerular filtration rate; MMF, mycophenolate mofetil; MR, multiple relapses; NR, no relapse; PRED, prednisolone. Categorical variables were expressed as frequency (percentage), and analyzed by the Chi square test. Continuous variables were expressed as mean ± SD, and analyzed with the Mann-Whitney test. ^†^Comparison between MR and NR.*

### Lymphocyte Subset Profiles in Lupus Nephritis Patients With Multiple Relapses or Those With No Relapse

Circulating naïve B cells were significantly lower in MR patients (median 0.7%, range 0.1–14.1%) compared with NR patients (median 4.0%, range 0.4–24.6%) (*p* = 0.017) (Table 2). The percentage of circulating memory B cells and plasma cells were comparable between the two groups. MR patients showed significantly higher memory-to-naïve B cell ratio [median (range): 0.8 (0.1–9.0), vs 0.2 (0.1–2.0) in NR patients, *p* = 0.024], and also numerically higher plasma cell-to-naïve B cell ratio [median (range): 0.3 (0.1–7.5), vs 0.1 (0.1–2.7) in NR patients, *p* = 0.098]. The percentage of circulating T cell subsets were similar between both groups (*p* > 0.05, for all) (Table 2).

**TABLE 2:** Circulating lymphocyte subset and cytokine profiles in lupus nephritis patients with multiple relapses or those with no relapse.

**Circulating lymphocyte subsets**	**MR (*n* = 19)**	**NR (*n* = 14)**	***p* value**
B lymphocyte subsets			
Naïve B cell (%)	0.7% (0.1–14.1%)	4.0% (0.4–24.6%)	0.017
Memory B cell (%)	0.6% (0.2–6.3%)	1.0% (0.1–3.2%)	0.123
Plasma cells (%)	0.2% (0.1–1.1%)	0.3% (0.1–2.9%)	0.382
Memory B/Naïve B ratio	0.8 (0.1–9.0)	0.2 (0.1–2.0)	0.024
Plasma cell/Naïve B cell ratio	0.3 (0.1–7.5)	0.1 (0.1–2.7)	0.098
T lymphocyte subsets			
CD8 + cytotoxic T cells (%)	25.8% (5.2–52.7%)	23.8% (8.2–48.9%)	0.808
Th1 cells (%)	3.3% (0.2–11.2%)	3.8% (0.1–6.4%)	1.000
Th2 cells (%)	3.9% (0.1–31.6%)	11.3% (0.2–47.7%)	0.087
Th17 cells (%)	0.3% (0.1–3.3%)	0.4% (0.1–2.2%)	0.545
Treg (%)	0.7% (0.1–3.0%)	0.7% (0.1–12.2%)	0.662
Serum cytokine levels			
BAFF (pg/ml)	1534.8 ± 674.3	1989.5 ± 2442.8	0.465
IL-6 (pg/ml)	445.1 ± 525.2	545.7 ± 740.6	0.708
IL-21 (pg/ml)	16.5 ± 22.7	19.2 ± 23.3	0.796
IFN-α (pg/ml)	29.4 ± 17.2	35.7 ± 27.7	0.781
IFN-γ (pg/ml)	125.8 ± 190.8	95.3 ± 106.1	0.679
IL-2 (pg/ml)	3240.4 ± 3786.5	7050.4 ± 11487.4	0.464
IL-4 (pg/ml)	36.4 ± 10.6	39.5 ± 19.2	0.760
IL-10 (pg/ml)	8.5 ± 7.9	32.5 ± 83.1	0.707
IL-17 (pg/ml)	232.7 ± 246.2	238.5 ± 301.9	0.679
IL-18 (pg/ml)	230.3 ± 127.1	238.0 ± 166.8	0.674
IL-23 (pg/ml)	13.3 ± 3.4	25.3 ± 41.1	0.947

*MR, multiple relapses; NR, no relapse; Th, T helper cells; Treg, regulatory T cells; BAFF, B cell activating factor; IFN, interferon; IL, interleukin. Results expressed as median (range) or mean ± SD, and analyzed with the Mann-Whitney test.*

### Relationship Between Lymphocyte Subsets, and Anti-dsDNA Antibodies and C3 Levels in Lupus Nephritis Patients With Multiple Relapses or Those With No Relapse

Lymphocyte subsets showed no relationship with serum anti-dsDNA antibodies and C3 levels during disease quiescence in MR and NR patients (*p* > 0.05, for all).

### Serum Cytokine Profiles in Lupus Nephritis Patients With Multiple Relapses or Those With No Relapse

The MR and NR patients did not differ in their serum levels of BAFF, IL-2, IL-4, IL-6, IL-10, IL-17, IL-18, IL-21, IL-23, IFN-α, and IFN-γ during disease quiescence (*p* > 0.05, for all) (Table 2).

### Serum miR-148a Expression in Lupus Nephritis Patients With Multiple Relapses or Those With No Relapse

The MR group showed significantly higher serum miR-148a expression than the NR group (1.0 ± 0.0, 0.7 ± 0.2, and 9.4 ± 6.9 fold difference for healthy controls (HC) and NR and MR patients respectively; *p* < 0.001, MR vs NR or HC).

### miR-148a, BACH1, BACH2, and PAX5 Expression in Naïve B Cells From Lupus Nephritis Patients With Multiple Relapses or Those With No Relapse

**FIGURE 2:**
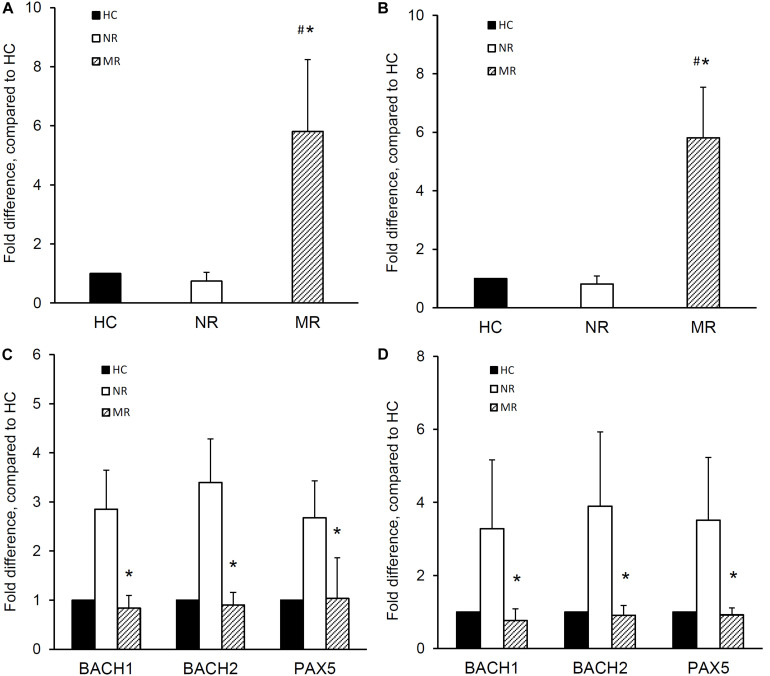
miR-148a, BACH1, BACH2, and PAX5 expression in naïve and memory B cells isolated from lupus nephritis patients with multiple relapses or those with no relapse. Naïve and memory B cells were isolated from lupus nephritis patients with multiple relapses (MR, 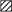) (*n* = 19) or those with no relapse (NR, 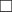) (*n* = 14). miR-148a and BACH1, BACH2, and PAX5 expressions was determined by qPCR. miR-148a expression in panel **(A)** naïve B cells and **(B)** memory B cells was normalized to U6 expression and BACH1, BACH2, and PAX5 expressions in panel **(C)** naïve B cells and **(D)** memory B cells were normalized to GAPDH expression, and expressed as fold difference compared to healthy controls (HC, 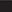) (*n* = 10). Data expressed as mean ± SD and analyzed by ANOVA followed by a *post hoc* Dunnett’s test. *p < 0.001, MR vs NR; ^#^*p* < 0.001, MR vs HC.

The expression of miR-148a, BACH1, BACH2, and PAX5 was determined in naïve B cells isolated from MR and NR patients. The miR-148a expression was significantly higher in the naïve B cells from MR patients compared with NR patients (1.0 ± 0.0, 0.7 ± 0.3, and 5.8 ± 2.4 fold difference for HC, NR, and MR respectively; *p* < 0.001, MR vs NR or HC) (Figure 2A). Naïve B cells from MR patients showed lower BACH1 (1.0 ± 0.0, 2.9 ± 0.8, and 0.8 ± 0.3 fold difference for HC, NR, and MR respectively), BACH2 (1.0 ± 0.0, 3.4 ± 0.9, and 0.9 ± 0.3 fold difference for HC, NR, and MR respectively), and PAX5 (1.0 ± 0.0, 2.7 ± 0.8, and 1.0 ± 0.8 fold difference for HC, NR, and MR respectively) expression compared with NR patients (*p* < 0.001, MR vs NR, for all) (Figure 2C).

### miR-148a, BACH1, BACH2, and PAX5 Expression in Memory B Cells From Lupus Nephritis Patients With Multiple Relapses or Those With No Relapse

The expression of miR-148a, BACH1, BACH2, and PAX5 was also determined in memory B cells from MR and NR patients. Memory B cells from MR patients showed significantly higher miRNA-148a expression than NR patients and HC (1.0 ± 0.0, 0.8 ± 0.3, and 5.8 ± 1.7 fold difference for HC, NR, and MR respectively; *p* < 0.001, MR vs NR or HC) (Figure 2B).

Memory B cells from MR patients also showed significantly lower BACH1 expression than NR patients (1.0 ± 0.0, 3.3 ± 1.9, and 0.8 ± 0.3 fold difference for HC, NR, and MR respectively; *p* < 0.001, MR vs NR). BACH2 and PAX5 expression was also lower in MR patients compared to NR patients (BACH2: 1.0 ± 0.0, 3.9 ± 2.0, and 0.9 ± 0.3 fold difference; PAX5: 1.0 ± 0.0, 3.5 ± 1.7, and 0.9 ± 0.2 fold difference for HC, NR, and MR respectively; *p* < 0.001, MR compared with NR, for both) (Figure 2D).

### Effect of Antagomir-148a Treatment on BACH1, BACH2, and PAX5 Expression and Cell Proliferation in Naïve and Memory B Cells

The effect of miR-148a on the BACH1, BACH2, and PAX5 expression and cell proliferation was next investigated in naïve and memory B cells isolated from eight treatment-naïve active LN patients (Table 1).

**FIGURE 3:**
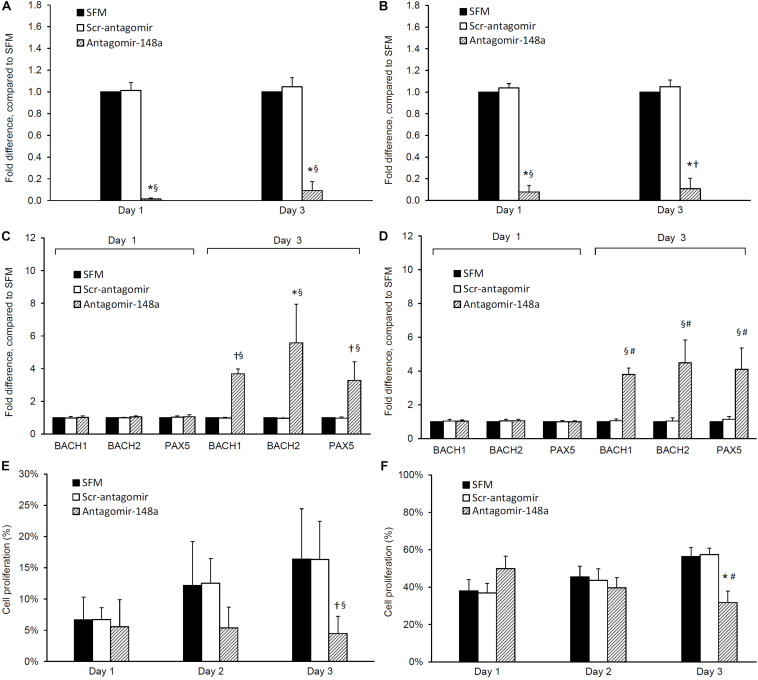
Effect of antagomir-148a on miRNA-148a, BACH1, BACH2, and PAX5 expression and cell proliferation in naïve and memory B cells from treatment-naïve active lupus nephritis patients. Naïve and memory B cells were isolated from eight treatment-naïve active lupus nephritis patients and cultured with serum free medium (SFM) (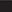) in the presence or absence of scrambled antagomir sequence (Scr-antagomir) (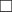) or antagomir-148a (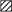) for 2 h. Naïve and memory B cells were then stimulated with CpG (in RPMI 1640 medium containing 10% FCS and 1% penicillin/streptomycin) for 3 days, total RNA extracted and miR-148a, BACH1, BACH2, and PAX5 expressions determined by qPCR. miR-148a expression in panels **(A)** naïve and **(B)** memory B cells were normalized to U6 expression, and BACH1, BACH2, and PAX5 expression in panels **(C)** naïve B cells and **(D)** memory B cells were normalized to GAPDH expression respectively, and expressed as fold difference compared to SFM. Cell proliferation in panels **(E)** naïve B cells and **(F)** memory B cells was determined by CSFE proliferation assay after 1, 2, and 3 days. Data expressed as mean ± SD and analyzed by ANOVA followed by a *post hoc* Dunnett’s test. *p < 0.001, ^§^*p* < 0.05, antagomir-148a vs Scr-Antagomir; ^#^*p* < 0.001, ^†^*p* < 0.05 antagomir-148a vs SFM.

The incubation of naïve B cells with antagomir-148a mitigated miR-148a expression after 24 h, and this inhibition was sustained for 3 days (*p* < 0.001, antagomir-148a vs Scr-antagomir or no treatment) (Figure 3A). After 3 days, the inhibition of miR-148a expression was accompanied by a 3.7 ± 0.3, 5.6 ± 2.6, and 3.3 ± 1.1 fold increase in BACH1, BACH2, and PAX5 expression respectively compared to control cells (*p* < 0.01 for all, antagomir-148a vs Scr-antagomir) (Figure 3C). Incubation of naïve B cells with antagomir-148a for 3 days significantly suppressed cell proliferation compared to cells incubated with Scr-antagomir (4.5 ± 2.8% vs 16.3 ± 8.0%, antagomir-148a vs Scr-antagomir, *p* < 0.01) (Figure 3E).

Similarly, incubation of memory B cells with antagomir-148a abrogated miR-148a expression after 24 h, and the inhibition was sustained for 3 days (Figure 3B). miRNA-148a inhibition was accompanied by a 3.8 ± 0.4, 4.5 ± 1.4, and 4.1 ± 1.3 fold increase in BACH1, BACH2, and PAX5 expression respectively compared to control cells (*p* < 0.01 for all, antagomir-148a vs Scr-antagomir) (Figure 3D). Inhibition of miR-148a expression significantly reduced cell proliferation compared to control cells (31.7 ± 6.2% vs 57.4 ± 3.4%, antagomir-148a vs Scr-antagomir, *p* < 0.001) (Figure 3F).

## Discussion

The B cell repertoire has crucial pathogenic roles in LN which include synthesis of autoantibodies, presentation of autoantigens, and secretion of pro-inflammatory and anti-inflammatory mediators that regulate the immune response (29–36). Accumulating evidence suggests that SLE is associated with perturbations in B lymphocyte subpopulations, epitomized by an expansion of class-switched memory B cells relative to naïve cells in active lupus patients compared to patients during disease quiescence (10, 37, 38). Changes in B cell subtypes pertaining to LN relapse, however, remain to be characterized.

Our current data revealed that, during disease remission, MR patients exhibited a higher memory-to-naïve B cell ratio and decreased circulating naïve B cells compared with NR patients. Alterations in the B cell subset profile in MR patients may be related to repeated exposure to autoantigens, which stimulates naïve B cells to differentiate into more mature B cell subtypes. Memory B cells have multiple properties pertinent to disease relapse in SLE and LN patients. B lymphocytes which differentiate into memory B cells typically remain dormant and are therefore less susceptible to conventional induction agents which show cell-cycle dependent anti-proliferative effects (38, 39). These memory B cells, nevertheless, possess long-lasting immunological memory and can generate potent and efficient immune response following stimulation with previously encountered antigens (39). Memory B cells from lupus patients also show reduced FcγRIIb expression, which leads to an augmented influx of calcium ion and diminished inhibitory signals for memory B cell activation (10, 40).

Whilst plasma cells are the key pathogenic cells responsible for autoantibody production in LN (10, 38), we did not find any significant difference in the number of circulating plasma cells between MR and NR patients. Detecting a difference in circulating plasma cells can be difficult because plasma cells predominantly reside in the bone marrow and are only present in very low frequencies in the circulation. Emerging data suggests the pathogenic roles of T lymphocytes in LN, with the CD4^+^ T helper subset being a major driver for B cell differentiation. Aberrant signaling, proliferation, and cytokine production have been observed in T cells from SLE patients (25, 41–45). Here we detect no significant difference in the T cell subsets profile between MR and NR patients, and it remains possible that functional rather than quantitative differences in T lymphocytes occur in MR and NR patients although such postulations need verification in further studies.

The homeostasis and immunological functions of B and T lymphocytes are regulated by various pro-inflammatory and anti-inflammatory cytokines such as BAFF, IL-2, IL-4, IL-6, IL-10, IL-17, IL-18, IL-21, IL-23, IFN-α, and IFN-γ (19–27). In this study, we also measured the levels of these cytokines to investigate whether their changes could affect the B cell subset profiles in MR and NR patients. We did not detect any change in the serum levels of these cytokines, and this may be attributed to the time of sample collection, that is, during disease remission.

miR-148a plays an instrumental role in B cell development, and its increased expression has been observed in B and T lymphocytes and also tissue pathologies in SLE patients and murine lupus models (46–49). Upregulated miR-148a expression renders B lymphocytes more resilient to B cell receptor-induced apoptosis, resulting in improved survival of immature B cells (14). BACH1 and BACH2 are essential transcription factors in B lymphocytes that are regulated by miR-148a, and together they exert suppressive effects on the maturation and homeostasis of B lymphcoytes (13–15, 17, 18). PAX5 has also been reported to inhibit the development and proliferation of B lymphocytes but its relationship with miR-148a remains poorly understood (16). Based on our findings in the B cell subset profile, we next evaluated the blood miR-148a level and its expressions in naïve and memory B cells, and also investigated the effect of miR-148a on BACH1, BACH2, and PAX5 expression in B lymphocytes. Our present data showed that, even during disease remission, MR patients had a significantly higher serum miR-148a level compared to NR patients. MR patients also exhibited higher miR-148a expression in naïve and memory B cells, and this was accompanied by lower BACH1, BACH2, and PAX5 expressions in B lymphocytes. To further elucidate the effect of miR-148a on BACH1, BACH2, and PAX5 expression and cell proliferation in naïve and memory B cells, we treated B cells isolated from active LN patients with antagomir-148a, an oligonucleotide that inhibits miR-148a expression. Inhibition of miR-148a resulted in an upregulation of BACH1, BACH2, and PAX5 expressed and decreased cell proliferation in naive and memory B cells, suggesting that miR-148a exerts an inhibitory effect on transcription factors which negatively modulates B lymphocyte differentiation and proliferation.

It has been shown that increased miR-148a expression can downregulate *Gadd45a*, *Bim*, and *PTEN* and inhibit apoptosis of immature B cells, thus leading to enhanced B cell autoreactivity (14). Reinstitution of miR-148a-transfected hematopoietic stem cells to lethally irradiated MRL/lpr mice restored lupus phenotypes and decreased the survival rates compared to control mice (14). Other investigators have demonstrated that miR-148a could downregulate BACH2 expression in B lymphocytes isolated from C57BL/6 mice, resulting in enhanced differentiation and survival of plasma cells (13). Over-expression of BACH2 would inhibit “myeloid genes” in pre- and pro-B cells, and thus divert them from committing to the lymphoid lineage (15). BACH2 can also interact with BCL-6 to suppress Blimp-1 expression during germinal center reaction, leading to reduced plasma cell differentiation (17). Independent researchers also reported that mouse splenic B cells deficient in BACH2 showed a more ready differentiation into plasma cells (18). Diminished BACH2 expression was observed in B lymphocytes from lupus patients, and transfection of BACH2 into these B cells resulted in increased apoptosis and suppressed proliferation (50). Furthermore, one recent GWAS meta-analysis also identified BACH2 as a susceptibility locus in Chinese lupus patients (51).

Data regarding the relationship between miR-148a and PAX5 expression is limited. Our results suggest that inhibition of miR-148a will upregulate PAX5 expression and suppresses B cell proliferation in LN patients. PAX5 is a master regulator in the development and function of B lymphocytes. During early B lymphocyte development, PAX5 can direct lymphoid progenitors to commit a B cell destiny by repressing “B-lineage-inappropriate” genes and simultaneously activating B-cell-specific genes (16). PAX5 can also induce V(H)-DJ(H) recombination during immunoglobulin production to enhance the antibody repertoire (16). In this study, we did not explore the interaction between BACH1, BACH2, and PAX5 in B lymphocytes. Notwithstanding, our findings suggest that elevated miR-148a expression in B lymphocytes from MR patients may decrease expression of BACH1, BACH2, and PAX5 and hence promote their differentiation into more mature B cell subpopulations. Such alterations in cellular signatures may account for the increased memory-to-naïve B cell ratio and decreased circulating naïve B cells as observed in MR patients, which thereby may increase propensity for disease relapse in LN patients. The mechanisms by which miR-148a downregulates BACH1, BACH2, and PAX5 expression remains obscure and putative mechanisms include base pairing to sequence motifs in the 3′UTR of mRNA with exact or closely analogous complementarities or epigenetic modifications of downstream target genes (46, 52, 53).

While it is important to conduct *in vivo* studies to verify our current *in vitro* findings, one should appreciate that the commonly used murine lupus models are not optimal for investigating disease relapse. Due to the limited clinical samples, we only measured the level of BACH1, BACH2, and PAX5

transcripts but not the protein levels, which remains an important shortcoming of this study. Another limitation of our study was that we did not examine the other immune-reactive cells and the complement cascades which are also highly relevant in LN pathogenesis. Also, clinical materials were only obtained during disease remission and were not compared with samples collected during active LN flares. Nevertheless, one should appreciate that patients in the MR and NR groups were carefully matched for patient characteristics and background immunosuppressive therapies, and thus the observed differences in B cell subsets and related cellular signatures is more likely due to intrinsic immunological abnormalities rather than patient heterogeneity and effect of treatments.

## Conclusion

The results suggest that altered B cell subsets and cellular signatures of miR-148a, BACH1, BACH2, and PAX5 are associated with distinct phenotypes related to the risk of renal relapse in patients with LN.

## Data Availability Statement

The raw data supporting the conclusions of this article will be made available by the authors, without undue reservation.

## Ethics Statement

The studies involving human participants were reviewed and approved by the Institutional Review Board of the University of Hong Kong/Hospital Authority Hong Kong West Cluster (Approval number: UW 12-389). The patients/participants provided their written informed consent to participate in this study.

## Author Contributions

DY, SY, and TC contributed to conception and conduct of the study, analysis of data and preparation of the manuscript. PL, IY, CTam, and CTang contributed to technical support and analysis of data. All authors critically revised and approved the final version of the manuscript and agreed to be accountable for all aspects of ensuring the accuracy and integrity of the work.

## Conflict of Interest

The authors declare that the research was conducted in the absence of any commercial or financial relationships that could be construed as a potential conflict of interest.

## References

[1] Yap DY Tang CS Ma MK Lam MF Chan TM. Survival analysis and causes of mortality in patients with lupus nephritis. *Nephrol Dial Transplant.* (2012) 27:3248–54. 10.1093/ndt/gfs073 22523116

[2] Bernatsky S Boivin JF Joseph L Manzi S Ginzler E Gladman DD Mortality in systemic lupus erythematosus. *Arthritis Rheum.* (2006) 54:2550–7. 10.1002/art.21955 16868977

[3] Moroni G Quaglini S Maccario M Banfi G Ponticelli C. “Nephritic flares” are predictors of bad long-term renal outcome in lupus nephritis. *Kidney Int.* (1996) 50:2047–53. 10.1038/ki.1996.528 8943489

[4] Parikh SV Nagaraja HN Hebert L Rovin BH. Renal flare as a predictor of incident and progressive CKD in patients with lupus nephritis. *Clin J Am Soc Nephrol.* (2014) 9:279–84. 10.2215/CJN.05040513 24262502 PMC3913239

[5] Mok CC Ho CT Siu YP Chan KW Kwan TH Lau CS Treatment of diffuse proliferative lupus glomerulonephritis: a comparison of two cyclophosphamide-containing regimens. *Am J Kidney Dis.* (2001) 38:256–64. 10.1053/ajkd.2001.26084 11479150

[6] Austin HA III Klippel JH Balow JE le Riche NG Steinberg AD Plotz PH Therapy of lupus nephritis. Controlled trial of prednisone and cytotoxic drugs. *N Engl J Med.* (1986) 314:614–9. 10.1056/NEJM198603063141004 3511372

[7] Illei GG Austin HA Crane M Collins L Gourley MF Yarboro CH Combination therapy with pulse cyclophosphamide plus pulse methylprednisolone improves long-term renal outcome without adding toxicity in patients with lupus nephritis. *Ann Intern Med.* (2001) 135:248–57. 10.7326/0003-4819-135-4-200108210-00009 11511139

[8] Yap DYH Chan TM. B cell abnormalities in systemic lupus erythematosus and lupus nephritis-role in pathogenesis and effect of immunosuppressive treatments. *Int J Mol Sci.* (2019) 20:6231. 10.3390/ijms20246231 31835612 PMC6940927

[9] Yap DY Lai KN. The role of cytokines in the pathogenesis of systemic lupus erythematosus – from bench to bedside. *Nephrology.* (2013) 18:243–55. 10.1111/nep.12047 23452295

[10] Dorner T Jacobi AM Lee J Lipsky PE. Abnormalities of B cell subsets in patients with systemic lupus erythematosus. *J Immunol Methods.* (2011) 363:187–97. 10.1016/j.jim.2010.06.009 20598709

[11] Tiller T Tsuiji M Yurasov S Velinzon K Nussenzweig MC Wardemann H. Autoreactivity in human IgG+ memory B cells. *Immunity.* (2007) 26:205–13. 10.1016/j.immuni.2007.01.009 17306569 PMC1839941

[12] Anolik JH Barnard J Cappione A Pugh-Bernard AE Felgar RE Looney RJ Rituximab improves peripheral B cell abnormalities in human systemic lupus erythematosus. *Arthritis Rheum.* (2004) 50:3580–90. 10.1002/art.20592 15529346

[13] Porstner M Winkelmann R Daum P Schmid J Pracht K Côrte-Real J miR-148a promotes plasma cell differentiation and targets the germinal center transcription factors Mitf and Bach2. *Eur J Immunol.* (2015) 45:1206–15. 10.1002/eji.201444637 25678371

[14] Gonzalez-Martin A Adams BD Lai M Shepherd J Salvador-Bernaldez M Salvador JM The microRNA miR-148a functions as a critical regulator of B cell tolerance and autoimmunity. *Nat Immunol.* (2016) 17:433–40. 10.1038/ni.3385 26901150 PMC4803625

[15] Itoh-Nakadai A Hikota R Muto A Kometani K Watanabe-Matsui M Sato Y The transcription repressors Bach2 and Bach1 promote B cell development by repressing the myeloid program. *Nat Immunol.* (2014) 15:1171–80. 10.1038/ni.3024 25344725

[16] Medvedovic J Ebert A Tagoh H Busslinger M. Pax5: a master regulator of B cell development and leukemogenesis. *Adv Immunol.* (2011) 111:179–206. 10.1016/B978-0-12-385991-4.00005-2 21970955

[17] Huang C Geng H Boss I Wang L Melnick A. Cooperative transcriptional repression by BCL6 and BACH2 in germinal center B-cell differentiation. *Blood.* (2014) 123:1012–20. 10.1182/blood-2013-07-518605 24277074 PMC3924924

[18] Muto A Ochiai K Kimura Y Itoh-Nakadai A Calame KL Ikebe D Bach2 represses plasma cell gene regulatory network in B cells to promote antibody class switch. *EMBO J.* (2010) 29:4048–61. 10.1038/emboj.2010.257 20953163 PMC3020649

[19] Muraguchi A Hirano T Tang B Matsuda T Horii Y Nakajima K The essential role of B cell stimulatory factor 2 (BSF-2/IL-6) for the terminal differentiation of B cells. *J Exp Med.* (1988) 167:332–44. 10.1084/jem.167.2.332 3258006 PMC2188837

[20] Terrier B Costedoat-Chalumeau N Garrido M Geri G Rosenzwajg M Musset L Interleukin 21 correlates with T cell and B cell subset alterations in systemic lupus erythematosus. *J Rheumatol.* (2012) 39:1819–28. 10.3899/jrheum.120468 22859347

[21] Cassese G Arce S Hauser AE Lehnert K Moewes B Mostarac M Plasma cell survival is mediated by synergistic effects of cytokines and adhesion-dependent signals. *J Immunol.* (2003) 171:1684–90. 10.4049/jimmunol.171.4.1684 12902466

[22] Mackay F Woodcock SA Lawton P Ambrose C Baetscher M Schneider P Mice transgenic for BAFF develop lymphocytic disorders along with autoimmune manifestations. *J Exp Med.* (1999) 190:1697–710. 10.1084/jem.190.11.1697 10587360 PMC2195729

[23] Linker-Israeli M Bakke AC Kitridou RC Gendler S Gillis S Horwitz DA. Defective production of interleukin 1 and interleukin 2 in patients with systemic lupus erythematosus (SLE). *J Immunol.* (1983) 130:2651–5. 6222112

[24] Mosmann TR Cherwinski H Bond MW Giedlin MA Coffman RL. Two types of murine helper T cell clone. I. Definition according to profiles of lymphokine activities and secreted proteins. *J Immunol.* (1986) 136:2348–57. 2419430

[25] Akahoshi M Nakashima H Tanaka Y Kohsaka T Nagano S Ohgami E Th1/Th2 balance of peripheral T helper cells in systemic lupus erythematosus. *Arthritis Rheum.* (1999) 42:1644–8. 10.1002/1529-0131(199908)42:8<1644::AID-ANR12>3.0.CO;2-L 10446863

[26] Langrish CL Chen Y Blumenschein WM Mattson J Basham B Sedgwick JD IL-23 drives a pathogenic T cell population that induces autoimmune inflammation. *J Exp Med.* (2005) 201:233–40. 10.1084/jem.20041257 15657292 PMC2212798

[27] Liu X Bao C Hu D. Elevated interleukin-18 and skewed Th1:Th2 immune response in lupus nephritis. *Rheumatol Int.* (2012) 32:223–9. 10.1007/s00296-010-1609-9 20963419

[28] Eickenberg S Mickholz E Jung E Nofer JR Pavenstadt HJ Jacobi AM. Mycophenolic acid counteracts B cell proliferation and plasmablast formation in patients with systemic lupus erythematosus. *Arthritis Res Ther.* (2012) 14:R110. 10.1186/ar3835 22571761 PMC4060361

[29] Sekine H Watanabe H Gilkeson GS. Enrichment of anti-glomerular antigen antibody-producing cells in the kidneys of MRL/MpJ-Fas(lpr) mice. *J Immunol.* (2004) 172:3913–21. 10.4049/jimmunol.172.6.3913 15004199

[30] Espeli M Bokers S Giannico G Dickinson HA Bardsley V Fogo AB Local renal autoantibody production in lupus nephritis. *J Am Soc Nephrol.* (2011) 22:296–305. 10.1681/ASN.2010050515 21088295 PMC3029902

[31] Chan OT Hannum LG Haberman AM Madaio MP Shlomchik MJ. A novel mouse with B cells but lacking serum antibody reveals an antibody-independent role for B cells in murine lupus. *J Exp Med.* (1999) 189:1639–48. 10.1084/jem.189.10.1639 10330443 PMC2193634

[32] Chan OT Madaio MP Shlomchik MJ. B cells are required for lupus nephritis in the polygenic, Fas-intact MRL model of systemic autoimmunity. *J Immunol.* (1999) 163:3592–6. 10490951

[33] Luo J Niu X Liu H Zhang M Chen M Deng S. Up-regulation of transcription factor Blimp1 in systemic lupus erythematosus. *Mol Immunol.* (2013) 56:574–82. 10.1016/j.molimm.2013.05.241 23911415

[34] Guimaraes PM Scavuzzi BM Stadtlober NP Franchi Santos LFDR Lozovoy MAB Iriyoda TMV Cytokines in systemic lupus erythematosus: far beyond Th1/Th2 dualism lupus: cytokine profiles. *Immunol Cell Biol.* (2017) 95:824–31. 10.1038/icb.2017.53 28649995

[35] Salazar-Camarena DC Ortiz-Lazareno PC Cruz A Oregon-Romero E Machado-Contreras JR Muñoz-Valle JF Association of BAFF, APRIL serum levels, BAFF-R, TACI and BCMA expression on peripheral B-cell subsets with clinical manifestations in systemic lupus erythematosus. *Lupus.* (2016) 25:582–92. 10.1177/0961203315608254 26424128

[36] Malkiel S Barlev AN Atisha-Fregoso Y Suurmond J Diamond B. Plasma cell differentiation pathways in systemic lupus erythematosus. *Front Immunol.* (2018) 9:427. 10.3389/fimmu.2018.00427 29556239 PMC5845388

[37] Zhu L Yin Z Ju B Zhang J Wang Y Lv X Altered frequencies of memory B cells in new-onset systemic lupus erythematosus patients. *Clin Rheumatol.* (2018) 37:205–12. 10.1007/s10067-017-3877-1 29067587

[38] Odendahl M Jacobi A Hansen A Feist E Hiepe F Burmester GR Disturbed peripheral B lymphocyte homeostasis in systemic lupus erythematosus. *J Immunol.* (2000) 165:5970–9. 10.4049/jimmunol.165.10.5970 11067960

[39] Kurosaki T Kometani K Ise W. Memory B cells. *Nat Rev Immunol.* (2015) 15:149–59. 10.1038/nri3802 25677494

[40] Mackay M Stanevsky A Wang T Aranow C Li M Koenig S Selective dysregulation of the FcgammaIIB receptor on memory B cells in SLE. *J Exp Med.* (2006) 203:2157–64. 10.1084/jem.20051503 16923849 PMC2118390

[41] Masutani K Akahoshi M Tsuruya K Tokumoto M Ninomiya T Kohsaka T Predominance of Th1 immune response in diffuse proliferative lupus nephritis. *Arthritis Rheum.* (2001) 44:2097–106. 10.1002/1529-0131(200109)44:9<2097::AID-ART360>3.0.CO;2-6 11592372

[42] Masutani K Taniguchi M Nakashima H Yotsueda H Kudoh Y Tsuruya K Up-regulated interleukin-4 production by peripheral T-helper cells in idiopathic membranous nephropathy. *Nephrol Dial Transplant.* (2004) 19:580–6. 10.1093/ndt/gfg572 14767012

[43] Xing Q Wang B Su H Cui J Li J. Elevated Th17 cells are accompanied by FoxP3+ Treg cells decrease in patients with lupus nephritis. *Rheumatol Int.* (2012) 32:949–58. 10.1007/s00296-010-1771-0 21243492

[44] Dolff S Quandt D Wilde B Feldkamp T Hua F Cai X Increased expression of costimulatory markers CD134 and CD80 on interleukin-17 producing T cells in patients with systemic lupus erythematosus. *Arthritis Res Ther.* (2010) 12:R150. 10.1186/ar3100 20653937 PMC2945048

[45] Chen DY Chen YM Wen MC Hsieh TY Hung WT Lan JL. The potential role of Th17 cells and Th17-related cytokines in the pathogenesis of lupus nephritis. *Lupus.* (2012) 21:1385–96. 10.1177/0961203312457718 22892208

[46] Pan W Zhu S Yuan M Cui H Wang L Luo X MicroRNA-21 and microRNA-148a contribute to DNA hypomethylation in lupus CD4+ T cells by directly and indirectly targeting DNA methyltransferase 1. *J Immunol.* (2010) 184:6773–81. 10.4049/jimmunol.0904060 20483747

[47] Te JL Dozmorov IM Guthridge JM Nguyen KL Cavett JW Kelly JA Identification of unique microRNA signature associated with lupus nephritis. *PLoS One.* (2010) 5:e10344. 10.1371/journal.pone.0010344 20485490 PMC2867940

[48] Stagakis E Bertsias G Verginis P Nakou M Hatziapostolou M Kritikos H Identification of novel microRNA signatures linked to human lupus disease activity and pathogenesis: miR-21 regulates aberrant T cell responses through regulation of PDCD4 expression. *Ann Rheum Dis.* (2011) 70:1496–506. 10.1136/ard.2010.139857 21602271

[49] Chauhan SK Singh VV Rai R Rai M Rai G. Differential microRNA profile and post-transcriptional regulation exist in systemic lupus erythematosus patients with distinct autoantibody specificities. *J Clin Immunol.* (2014) 34:491–503. 10.1007/s10875-014-0008-5 24659206

[50] Zhu Z Yang C Wen L Liu L Zuo X Zhou F Bach2 regulates aberrant activation of B cell in systemic lupus erythematosus and can be negatively regulated by BCR-ABL/PI3K. *Exp Cell Res.* (2018) 365:138–44. 10.1016/j.yexcr.2018.02.034 29501569

[51] Morris DL Sheng Y Zhang Y Wang YF Zhu Z Tombleson P Genome-wide association meta-analysis in Chinese and European individuals identifies ten new loci associated with systemic lupus erythematosus. *Nat Genet.* (2016) 48:940–6. 10.1038/ng.3603 27399966 PMC4966635

[52] Lai EC. Micro RNAs are complementary to 3′ UTR sequence motifs that mediate negative post-transcriptional regulation. *Nat Genet.* (2002) 30:363–4. 10.1038/ng865 11896390

[53] Xie X Lu J Kulbokas EJ Golub TR Mootha V Lindblad-Toh K Systematic discovery of regulatory motifs in human promoters and 3′ UTRs by comparison of several mammals. *Nature.* (2005) 434:338–45. 10.1038/nature03441 15735639 PMC2923337

